# Supplementary figures and images for: A Wnt-BMP4 Signaling Axis Induces MSX and NOTCH Proteins and Promotes Growth Suppression and Differentiation in Neuroblastoma
Source: Cells. 2020 Mar 23;9(3):783. doi: 10.3390/cells9030783 (PMC7140810; doi:10.3390/cells9030783)

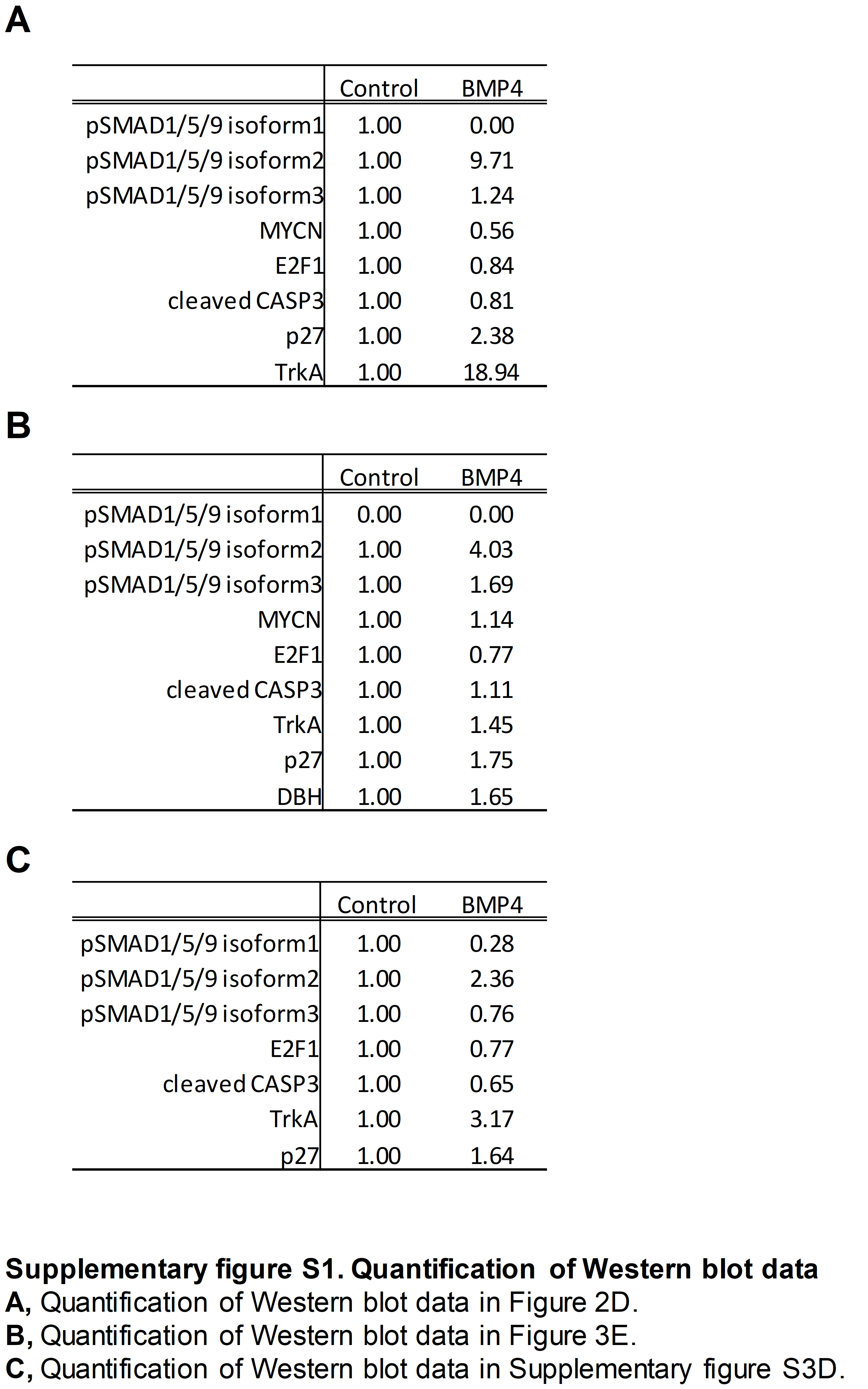

Supplement: Supplementary file 1 [file cells-09-00783-s001.zip › Supplementary figure S1.jpg]

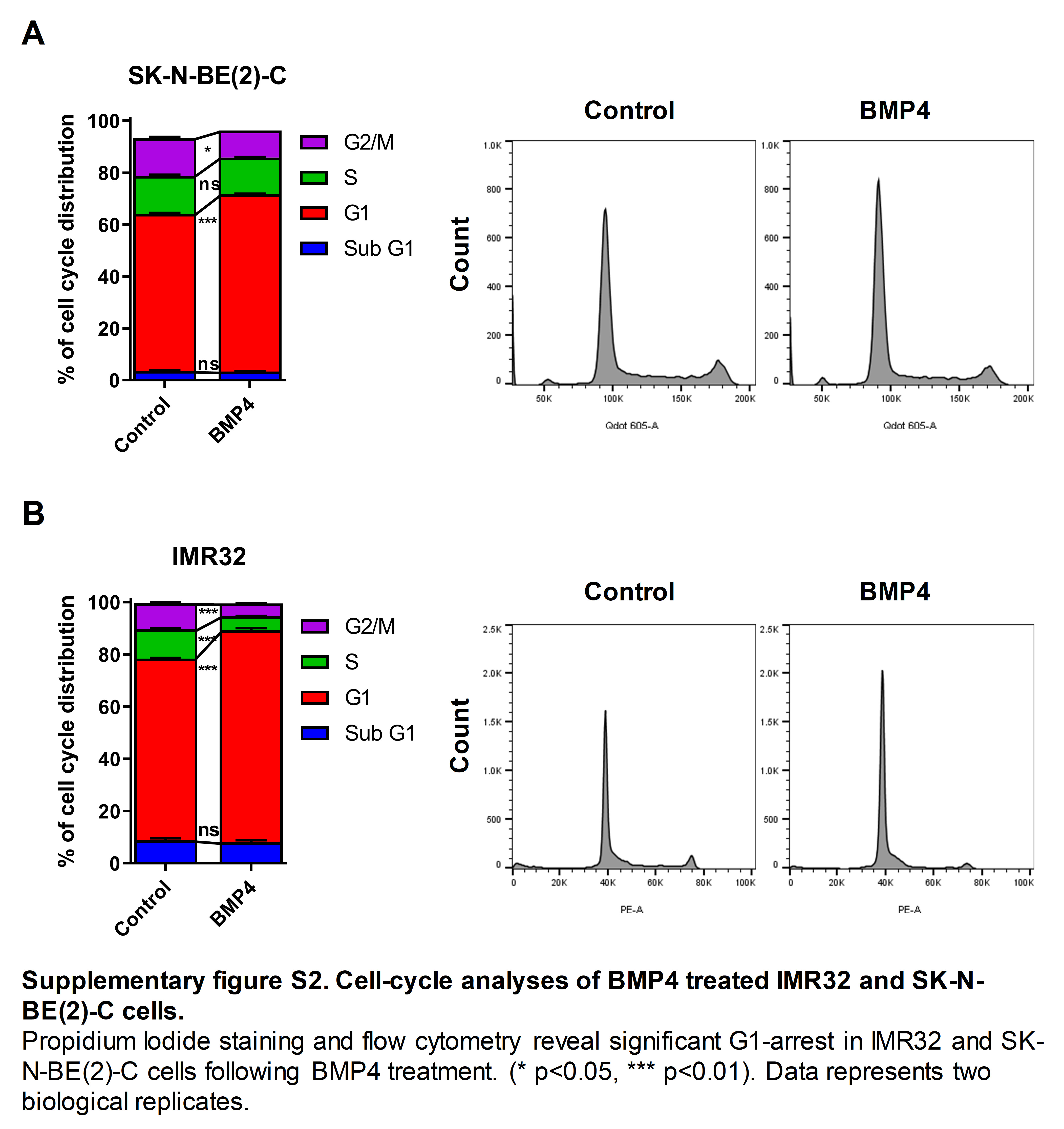

Supplement: Supplementary file 1 [file cells-09-00783-s001.zip › Supplementary figure S2.jpg]

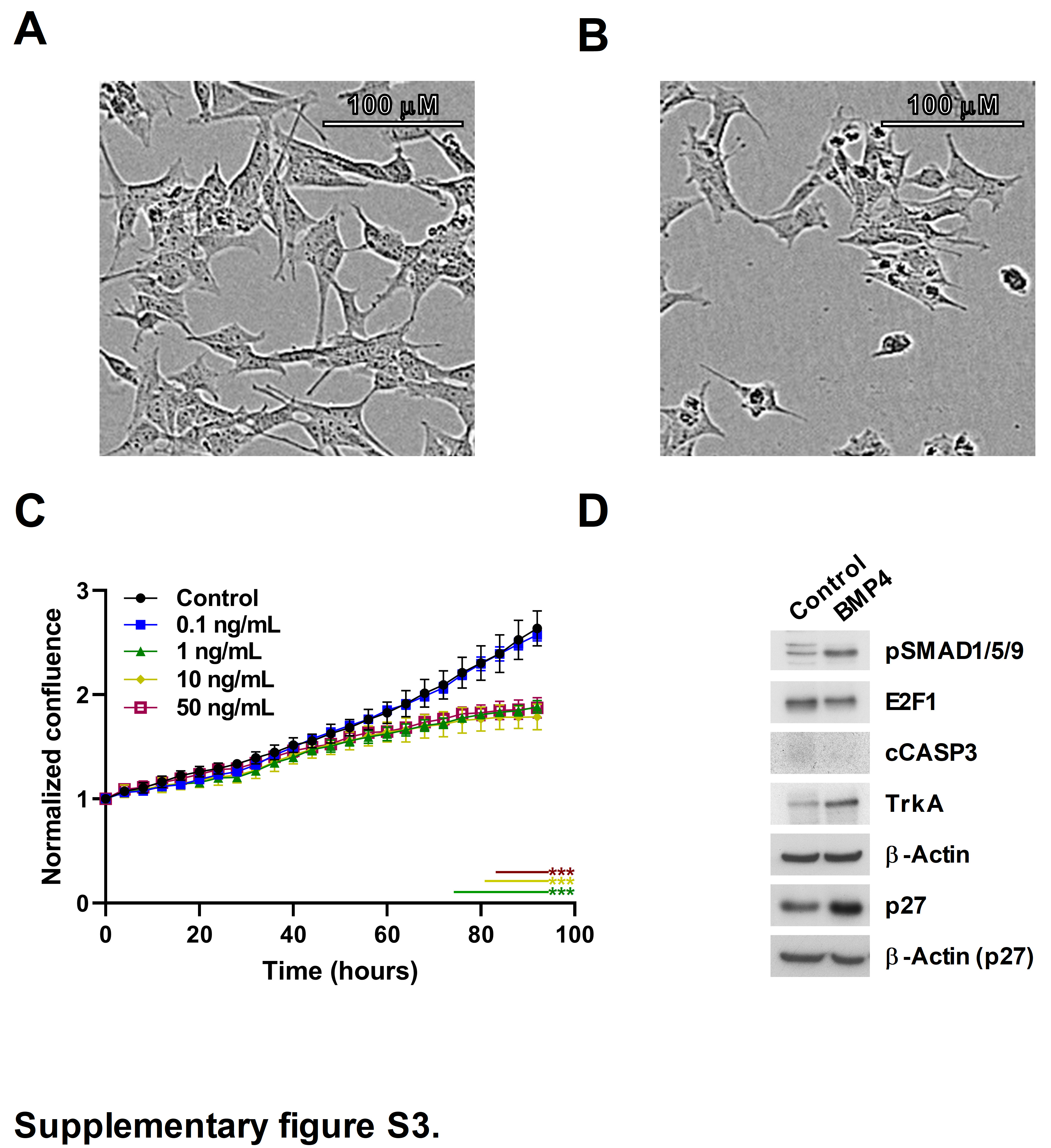

Supplement: Supplementary file 1 [file cells-09-00783-s001.zip › Supplementary figure S3.jpg]

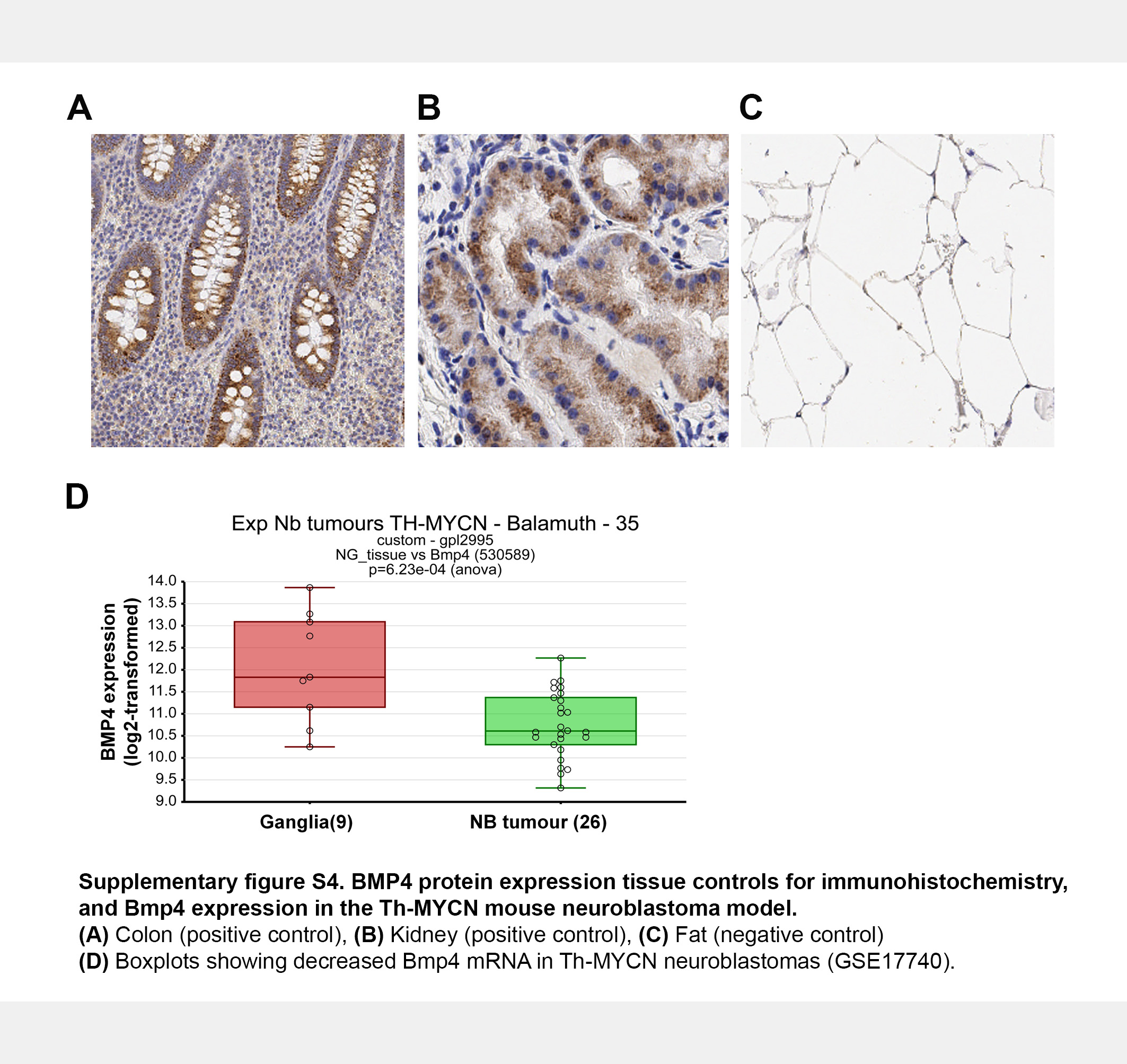

Supplement: Supplementary file 1 [file cells-09-00783-s001.zip › Supplementary figure S4.jpg]

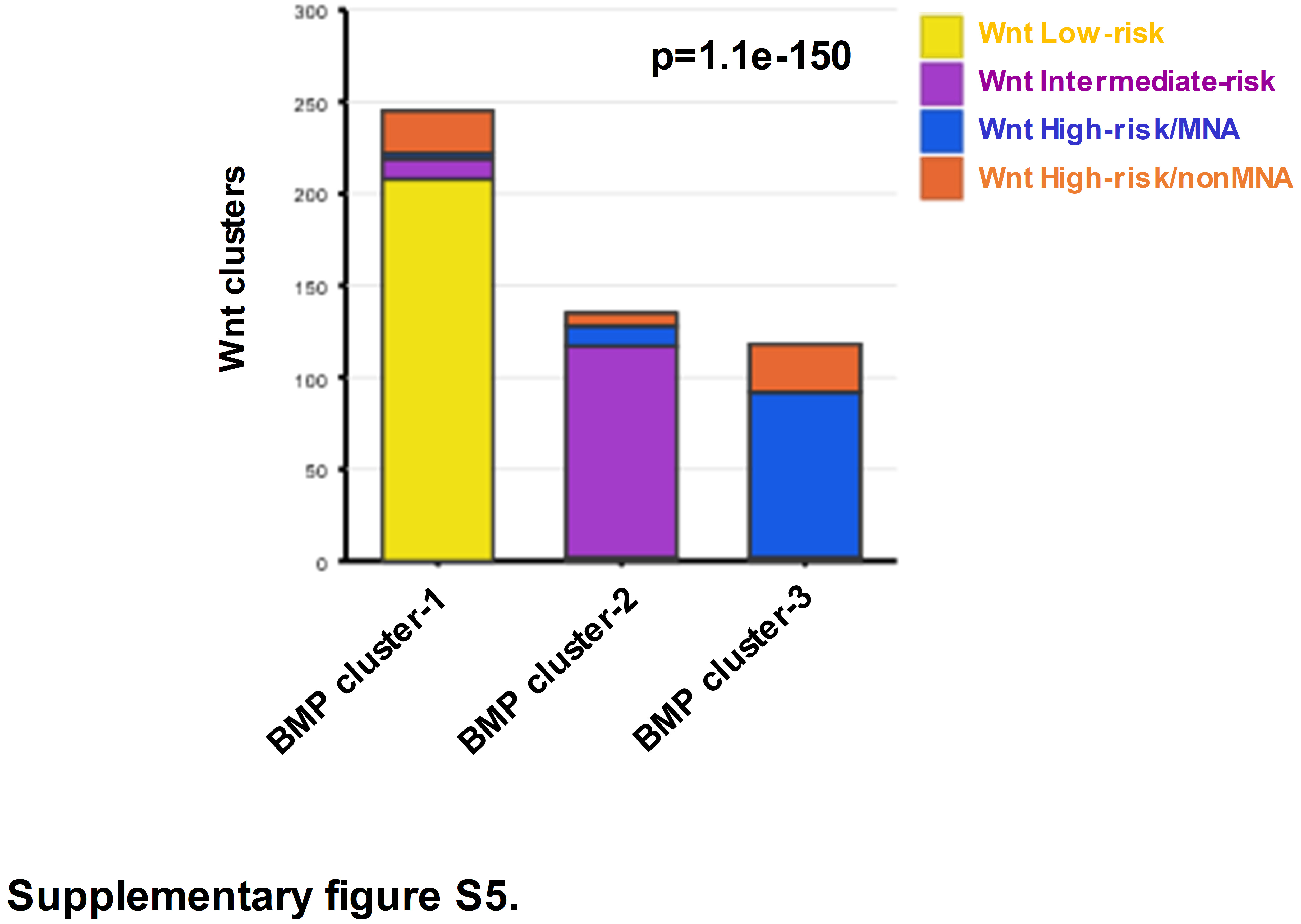

Supplement: Supplementary file 1 [file cells-09-00783-s001.zip › Supplementary figure S5.jpg]

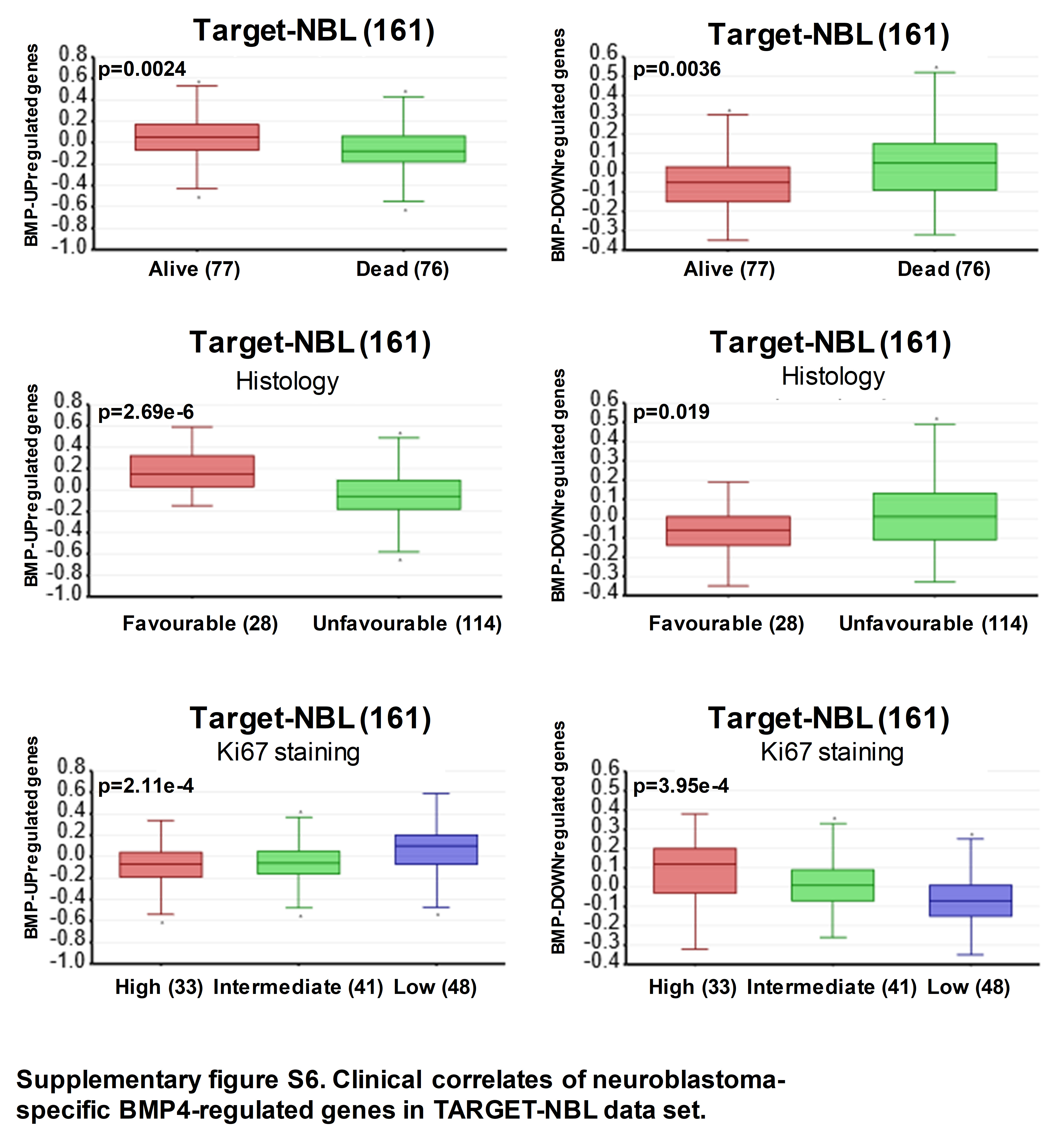

Supplement: Supplementary file 1 [file cells-09-00783-s001.zip › Supplementary figure S6.jpg]
